# Hypothesis: Nutrient Off-Loading and Ectopic Fat Reduction Reverse Insulin Resistance and Improve Cardiovascular Outcomes in Type 2 Diabetes—A Narrative Review

**DOI:** 10.3390/ijms27052150

**Published:** 2026-02-25

**Authors:** Joseph A. M. J. L. Janssen

**Affiliations:** Department of Internal Medicine, Erasmus Medical Center, Dr. Molewaterplein 40, 3015 GD Rotterdam, The Netherlands; j.a.m.j.l.janssen@erasmusmc.nl; Tel.: +31-06-1275-2413

**Keywords:** insulin resistance, hyperinsulinemia, type 2 diabetes, cardiovascular disease, Western diet, nutritional overload, physical inactivity, toxic fuel overload, metabolic stress, ectopic fat, superoxide, bariatric surgery, GLP-1 receptor agonists, SGLT2 inhibitors, nutrient off-loading, blood glucose control

## Abstract

Insulin resistance in type 2 diabetes is associated with cardiovascular disease. Nutritional overload, hyperinsulinemia, and physical inactivity are the major etiological factors driving the development of insulin resistance. In an obesogenic environment, insulin resistance has been proposed to protect the body against toxic fuel overload, hyperinsulinemia-induced injury, and metabolic stress. Insulin resistance has been further hypothesized to defend the heart and blood vessels against fuel overload when an individual is chronically overeating. Recent landmark cardiovascular outcome trials in type 2 diabetes show major improvements in cardiovascular disease outcomes after treatment with GLP-1 receptor agonists or SGLT2 inhibitors. Bariatric surgery achieves even greater improvements in cardiovascular disease outcomes than treatments with these newer pharmacological agents. It had been previously predicted that glucose-lowering approaches that normalize whole-body energy balance have the greatest potential to improve cardiovascular outcomes in type 2 diabetes. This review hypothesizes that treatment with bariatric surgery, GLP-1 receptor agonists, or SGLT2 inhibitors lowers glucose and nutritional off-loading, normalizes whole-body energy balance, and reduces ectopic fat depositions. This plays a central role in the dramatic reduction in cardiovascular disease and the reversal of insulin resistance in type 2 diabetes, which are observed after these three treatments.

## 1. Introduction

**Figure 1:**
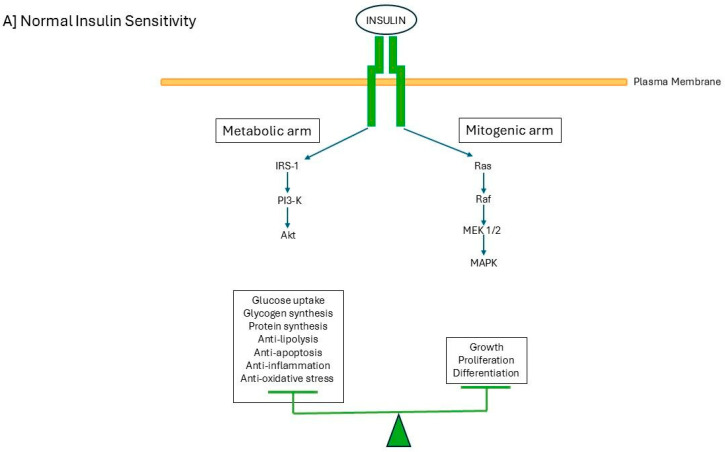

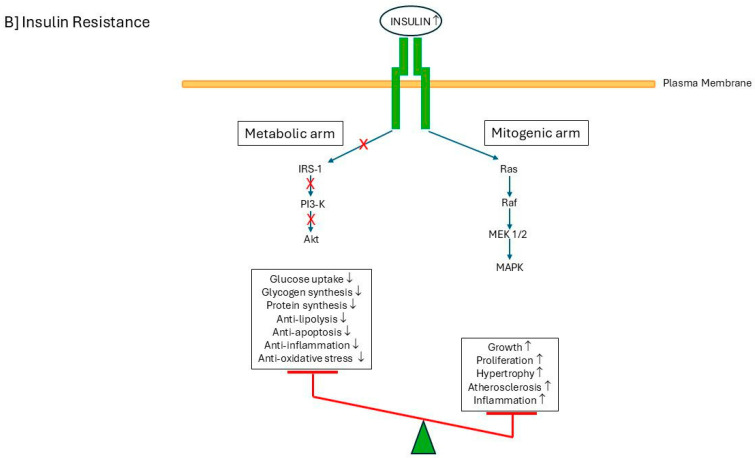
(**A**) The insulin receptor pathway in individuals with normal insulin receptor sensitivity. Activation of the insulin receptor results in parallel and balanced insulin signaling to the PI3KAkt pathway (metabolic arm) and the Ras-MAPK pathway (mitogenic arm). The metabolic pathway stimulates glucose uptake, glycogen synthesis, protein synthesis and inhibits lipolysis, apoptosis, inflammation and oxidative stress. Consequently, normal insulin receptor sensitivity is associated with normoglycemia and normal triglycerides. The mitogenic pathway stimulate growth, proliferation, and differentiation. In individuals with normal insulin receptor sensitivity insulin promotes a normal glucose metabolism, cell metabolism and a healthy cardiovascular system. (**B**) The insulin receptor pathway in individuals with insulin receptor resistance. Insulin resistance is characterized by a specific impairment in PI3K-dependent signaling pathway. This results in a decreased activation of the insulin receptor resulting in low glucose uptake, glycogen synthesis, protein synthesis and anti-lipolytic effects. Consequently, insulin resistance may cause hyperglycemia and hypertriglyceridemia. Simultaneously the unaffected mitogenic Ras-MAPK pathway is excessively stimulated and this may (in the long-term) contribute to cardiovascular disease by stimulating vascular smooth muscle cell proliferation, cellular migration and a prothrombotic state and accelerating atherosclerosis (modified from [5]; with permission). Abbreviations: IRS-1, insulin substrate receptor-1; PI3-K, phosphatidylinositol (PI)3-kinase; AKT: Protein Kinase B (PKB); Ras: rat sarcoma; Raf: rapidly accelerated fibrosarcoma; MEK, mitogen activated protein kinase; MAPK, mitogen-activated protein-kinase. X = decreased activation.

The insulin receptor is a heterotetramer formed by two extracellular α subunits, which bind insulin, and two membrane-spanning β subunits, each containing a tyrosine kinase domain. The binding of insulin to the insulin receptor on the plasma membrane is complex and affects multiple actions in the cell by increasing or decreasing various intracellular pathways [1]. Insulin binding causes the autophosphorylation of the insulin receptor, which, in turn, activates two main pathways downstream: the PI3K/AKT pathway (phosphatidylinositol 3-kinase/protein kinase B) and the MAPK pathway (mitogen-activated protein kinase) [2] (Figure 1). In individuals with normal insulin sensitivity, insulin-mediated effects of both signaling pathways are in balance (Figure 1A). However, in individuals with (relative) insulin resistance such as in type 2 diabetes, insulin-mediated signaling pathways are no longer in balance: the insulin-mediated downstream PI3K/AKT signaling is primarily affected, but the insulin-mediated downstream MAPK signaling is undisturbed (Figure 1B) [2]. Thus, insulin resistance in type 2 diabetes mainly affects insulin-mediated metabolic effects but not insulin-mediated mitogenic effects [2]. Unbalanced insulin receptor signaling causes impaired glucose uptake in typical insulin target tissues like the liver, skeletal muscle, and adipose tissues, but the hyperinsulinemia-mediated hyperactivation of the mitogen-activated protein kinase pathway may stimulate vascular smooth muscle cell proliferation, cellular migration, and a prothrombotic state (Figure 1). These effects can already be found long before people with insulin resistance clinically manifest abnormalities of glucose metabolism [3,4,5]. Hyperinsulinemia can shift the balance of insulin signaling towards a state that may lead to accelerated atherosclerosis [3,4,5]. Individuals with type 2 diabetes, hyperinsulinemia, and insulin resistance have an increased risk for cardiovascular disease (e.g., heart disease and stroke), which cannot be attributed to the classic cardiovascular risk factors [4,6]. In the short term, decreased insulin signaling limits the additional uptake of glucose. However, in the long term, chronically excess food intake and hyperinsulinemia can disrupt cellular homeostasis [7] (see below).

In this narrative review, papers were selected based on their relevance to build a coherent story by searching PubMed and Google Scholar.

## 2. What Is Insulin Resistance?

Insulin resistance is defined as a state in which normal insulin concentrations produce subnormal biological responses [8]. In clinical practice, insulin resistance is often referred to as a normal insulin concentration resulting in subnormal glucose lowering [9]. However, insulin resistance can be defined more widely and is not restricted to a single facet of insulin-mediated metabolism. Thus, insulin resistance can occur in relation to glucose metabolism and in relation to ion transport, protein synthesis, regulation of gene expression, etc. [9]. The intracellular molecular mechanisms producing insulin resistance can be caused by the decreased sensitivity of the insulin receptor to insulin, the decreased responsiveness of the insulin receptor to insulin, or a combination of both [8]. Disturbed interactions of insulin with the insulin receptor are more likely to produce states of decreased sensitivity [8]. In contrast, disruptions of the intracellular signaling pathways may primarily produce decreased responsiveness when the insulin receptor is stimulated with insulin [8].

The causes of insulin resistance in prediabetes and type 2 diabetes can be genetic and/or acquired [10]. The ‘Developmental Origins of Health and Disease hypothesis’ suggests that adverse events during intrauterine life may program during the early stages of fetal development for conditions such as insulin resistance, metabolic syndrome, and type 2 diabetes [11]. These conditions may clinically manifest later in life when there is an added impact of lifestyle and other conventionally acquired environmental risk factors (such as obesity, physical inactivity, and aging) interacting with genetic factors [11].

## 3. Major Causes of Insulin Resistance in the Population

Virtually all patients with type 2 diabetes show some degree of resistance to insulin-mediated actions on glucose metabolism. It is widely believed that genetic susceptibility is an important risk factor for developing insulin resistance. However, although Genome Wide Association Studies (GWASs) have identified thousands of genetic loci associated with insulin resistance and type 2 diabetes, by far, the majority of these genetic loci code for proteins involved in pancreatic β-cell function but not in the development of insulin resistance [12]. The etiology of insulin resistance may primarily have a genetic basis. However, the great majority of people have primarily acquired and secondary causes of insulin resistance, although genetic background may still play a role in this (Table 1) [13]. Many factors, conditions, and interactions may be involved in the development of acquired insulin resistance [13,14]. Nevertheless, to date, the exact molecular mechanisms of acquired insulin resistance are often not fully understood [13,14]. Overnutrition, hyperinsulinemia, glucose toxicity, and lipotoxicity are the important factors triggering insulin resistance [15].

## 4. Insulin Resistance Can Be a Transient Component of Normal Physiology

The development of insulin resistance during fasting/starvation is a normal physiological process that made survival possible throughout evolution in prolonged periods of famine [36]. Individuals show wide fluctuations in daily dietary intake and physical activity. The regulation of insulin sensitivity plays an important role in normal metabolic physiology. By changing insulin sensitivity, the body can regulate the distribution of ingested nutrients among different tissues [37]. For example, short-term caloric overfeeding results in a positive energy balance and induces skeletal muscle and liver insulin resistance after just 3 days of overfeeding [38]. The development of insulin resistance after short-term caloric overfeeding enables another distribution of nutrients: excess nutrients are transported to adipose tissue for storage [10]. In contrast, caloric restriction improves insulin sensitivity in skeletal muscles and the liver [39,40]. In humans, bariatric surgery and Very-Low-Calorie Diets (VLCDs) may rapidly induce improvements in liver insulin sensitivity by reducing the liver fat content. This improvement often occurs well before any substantial weight loss has occurred [41,42,43,44].

During fasting/starvation, there is a gradual depletion of fat stores [45]. Lipids in the subcutaneous fat stores are broken down (lipolysis), resulting in the release of glycerol and free fatty acids into the bloodstream [45]. The surge of free fatty acids in the bloodstream marks the shift from carbohydrate to lipid metabolism and leads to ectopic fat accumulation in the liver and skeletal muscle cells [36]. This directly results in plasma membrane diacylglycerol (DAG) build-up and protein kinase C activation, which induce insulin resistance in the liver and skeletal muscle cells [46]. This, in turn, preserves glucose in the bloodstream to fuel brain metabolism and other obligatory glucose-requiring cells in the body (e.g., red blood cells and renal medulla) [47].

Pregnancy is another condition that necessitates a temporarily altered diversion of maternal nutrients. During early gestation, insulin sensitivity of the mother increases and stimulates the uptake of glucose into adipose stores of the mother. It is thought that nutrients are stored to meet the feto-placental energy demands in early pregnancy and the maternal energy demands in late pregnancy and during lactation [48,49]. Therefore, early gestation is characterized by an anabolic state of the mother [49]. As pregnancy advances, insulin sensitivity decreases and insulin resistance develops [27]. In the 2nd and 3rd trimesters of pregnancies of healthy women, relative resistance in the mother to the actions of insulin on glucose uptake and utilization is a normal and expected physiological change [27]. Placental growth hormone, human placental lactogen, leptin, cortisol, progesterone, and estrogen may contribute to and be responsible for the development of insulin resistance during pregnancy [50]. The increase in insulin resistance in the mother during the 2nd and 3rd trimesters of pregnancy raises maternal glucose and free fatty acid concentrations. This ensures that the mother uses more fat than carbohydrates for energy and spares carbohydrates as a fuel for the fetus and maternal central nervous system [51]. The development of insulin resistance in pregnancy is thus a physiological adaptation of the mother to ensure adequate carbohydrate supply for the rapidly growing fetus [51]. For most women, resistance to the action of insulin disappears after childbirth as pregnancy hormones decrease and become normal again [51].

Rapid pubertal growth is another condition associated with a transient increase in insulin resistance [26]. Pubertal insulin resistance is also a normal physiological process. Insulin resistance starts at the onset of puberty (Tanner stage 2) but returns to near-prepubertal levels by the end of puberty (Tanner stage 5) [52]. The rise and fall of insulin resistance during puberty is regarded as necessary for puberty to proceed [53]. Puberty triggers a growth spurt, which increases nutritional needs [54]. Insulin resistance, along with compensating increased insulin secretion, has several effects on metabolism in puberty: it increases fat oxidation and decreases glucose oxidation [55]. It has been hypothesized that fuel metabolism during puberty is altered to preserve lean muscle mass and to maximize the availability of fat as an alternate fuel source [26]. Insulin resistance during puberty can be caused at least in part by changes in growth hormone secretion during puberty [56]. Growth hormone reaches a peak during puberty, and this peak declines at the end of puberty [57,58]. In addition, growth hormone can counteract the effects of insulin, primarily by promoting glucose production and inhibiting insulin’s action in the liver, muscles, and adipose tissue [59]. Therefore, growth hormone is considered a logical and major candidate for mediating a large part of pubertal insulin resistance [26]. In support of this, multiple studies have found a correlation between IGF-1, which is a marker of overall growth hormone secretion, and insulin sensitivity during puberty [55,60,61,62].

## 5. Is Insulin Resistance a Protective Response Against Insulin-Induced Metabolic Stress?

**Figure 2:**
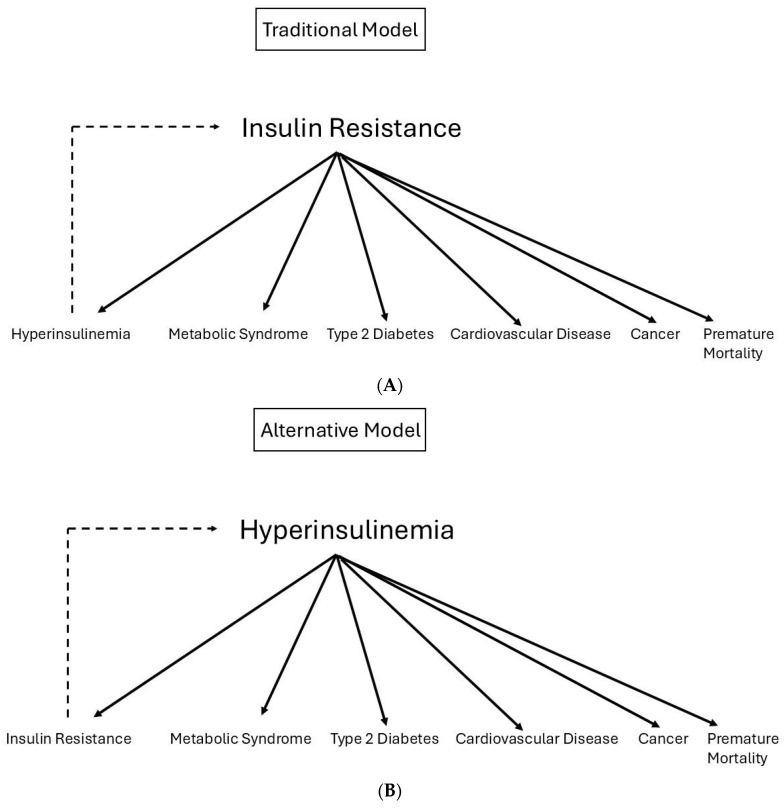
The traditional model vs. the alternative model. (**A**) In the traditional model, insulin resistance is the primary driver of metabolic syndrome, prediabetes, type 2 diabetes, cardiovascular disease, cancer, and premature mortality. Through feedback, hyperinsulinemia may further contribute to an increase in insulin resistance. (**B**) In recent years, a shift from this traditional insulin resistance-centered paradigm has occurred toward an alternative model in which hyperinsulinemia may act as a primary driver of metabolic disease. In the alternative model, hyperinsulinemia due to (over)nutrition, genetics, and/or the environment is the primary cause of secondary insulin resistance. Through feedback, insulin resistance may further contribute to an increase in hyperinsulinemia.

For many years, the prevailing view was that insulin resistance was the primary driver for metabolic syndrome, prediabetes, type 2 diabetes, cardiovascular disease, cancer, and premature mortality [63,64]. In this view, insulin resistance was considered as the primary and key culprit of these conditions, whereas hyperinsulinemia was thought to be the secondary culprit compensating for insulin resistance [37] (Figure 2A). However, in recent years, there has been growing evidence for an alternative concept. In this alternative concept, hyperinsulinemia is considered as causal in the development of insulin resistance and the primary driver for metabolic syndrome, prediabetes, type 2 diabetes, cardiovascular disease, cancer, and premature mortality [37,65,66,67] (Figure 2B). Hyperinsulinemia is thought to be caused by hyper-responsiveness of pancreatic β-cells to a hostile environment and drastic changes in lifestyle (Westernized diet, overnutrition, and a lack of physical activity) [37,65,66,67]. In vitro data demonstrated that pancreatic β-cells chronically exposed to excess nutrients indeed develop a hyper-response of insulin secretion to an acute glucose load at subnormal glucose concentrations [68]. However, a limitation is that to date the concept of β-cell hyper-responsiveness following chronic nutrient excess is mainly supported by in vitro and animal studies, which may not fully reflect the complexity of human metabolic regulation. In addition, future research should include longitudinal human studies and consider inter-individual metabolic heterogeneity, and it should be cautious when interpreting associations between hyperinsulinemia and cardiovascular disease/mortality. In addition, hyperinsulinemia and insulin resistance are closely intertwined biologically. Hyperinsulinemia and insulin resistance are likely to represent bidirectionally reinforcing processes, rather than a strictly one-way sequence; thus, hyperinsulinemia can be both a driver and a result of insulin resistance [16].

Reactive oxygen species (ROS) are highly reactive molecules that contain oxygen and are produced during normal cellular metabolism, particularly in the mitochondria [69]. Although ROS have a key role in normal cellular signaling, the excessive formation of ROS can cause cellular damage through a process called oxidative stress [70]. Basal hyperinsulinemia has been hypothesized to be a manifestation of underappreciated early pancreatic β-cell dysfunction, which is mediated by an interplay between increased oxidative stress and excess lipids in the form of a combination of ROS and long-chain acyl-CoA esters (LC-CoAs) [71]. Moreover, the production of ROS may be increased by inflammatory cytokines and certain exogenous environmental toxins [71].

In the alternative concept, chronic calorie surplus caused by overeating, physical inactivity, and hyperinsulinemia are the major etiological factors driving the development of insulin resistance, metabolic syndrome, prediabetes, type 2 diabetes, cardiovascular disease, cancer, and premature mortality [37,65,66,67] (Figure 2B).

Interestingly, insulin resistance has been proposed as a physiological defense mechanism against chronic fuel overload, nutrient/hyperinsulinemia-induced tissue injury, and metabolic stress [37,65,66,72,73,74]. A central component in this hypothesis is that excess substrate availability can itself induce insulin resistance and this impairs further substrate uptake to limit cellular substrate toxicity [73]. It was therefore hypothesized that the development of insulin resistance in certain tissues, in which insulin normally stimulates glucose entry (such as the liver, muscles, and fat tissue), protects these tissues from chronic hyperinsulinemia-mediated glucose excess (intracellular hyperglycemia) and glucose toxicity [37]. In the next paragraphs, potential mechanisms explaining the development of insulin resistance during fuel overload will be further discussed.

## 6. The Role of Western Diet and Ectopic Fat in Diacylglycerol-Mediated Insulin Resistance

The typical Western diet (high in calories, processed foods, and sugars) and chronic overnutrition lead to chronic hyperinsulinemia [75]. Hyperinsulinemia initially stimulates the storage of any fuel overload in the subcutaneous fat stores since this tissue has the highest capacity in the body to store fat [76]. The storage of fat in the subcutaneous tissue is a normal physiological process for the body to conserve energy for future (leaner) times. Individuals differ significantly in their ability to store fat in the subcutaneous fat tissue. Some individuals have an intrinsically greater capacity to store energy in the subcutaneous fat tissue, whereas other individuals have a considerably diminished capacity. Individual differences in subcutaneous fat storage capacity have been attributed to a combination of genetic, hormonal, and lifestyle factors [77]. When energy intake is chronically greater than energy expenditure, the maximum capacity of an individual’s subcutaneous fat cells to proliferate and store excessive substrate is exceeded at a certain moment. When the maximum capacity of the subcutaneous fat cells to store fat is exceeded, fatty acids will spillover. Free fatty acid spillover reflects the impaired insulin suppression of adipose tissue lipolysis and thus represents adipose tissue insulin resistance. Excessive fatty acids are transported to the liver, pancreas, heart, and skeletal muscles and are stored in these tissues as ectopic fat [75,77,78]. Ectopic fat depositions and its metabolites may be directly involved in the development of insulin resistance [75,77,78] (see below).

It has been suggested that impaired mitochondrial fat oxidation leads to increased ectopic fat depositions and insulin resistance [78,79]. Evidence in favor of this mechanism is derived from studies on the inherited defects in mitochondrial oxidative phosphorylation, which have been associated with insulin resistance [80]. For example, a 30% reduction in mitochondrial rates of adenosine triphosphate (ATP) synthesis, the energy-carrying molecule in living cells, and a 38% reduction in mitochondrial content were found in the muscles of young, lean, and sedentary insulin-resistant children of parents diagnosed with type 2 diabetes [80]. These children also had increased intramyocellular (ectopic) lipid content [80]. The increased intramyocellular lipid content has been hypothesized to be secondary to a low mitochondrial activity [81].

**Figure 3:**
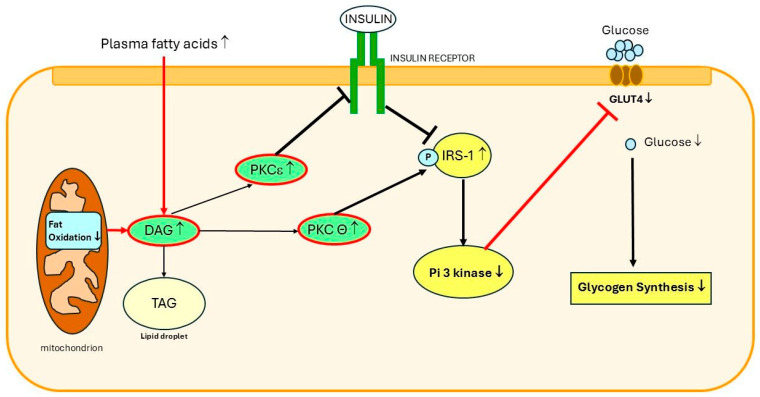
Molecular mechanism of ectopic lipid-induced muscle insulin resistance. An increased intramyocellular content of diacylglycerol (DAG), a metabolic intermediate of lipid metabolism, has been postulated to play a major role in causing insulin resistance. An increase in the DAG content is caused by an imbalance of intracellular fluxes: the rates of DAG synthesis (owing to increased fatty acid delivery and uptake into the skeletal muscle cells) exceed the rates of mitochondrial long-chain CoA oxidation and the incorporation of DAG into neutral lipids (triacylglycerol [TAG]) (which are stored in lipid droplets). The increased intracellular accumulation of DAG content results in the activation of the theta isoform of protein kinase C (PKC ϴ). The activation of PKC ϴ leads to the increased serine phosphorylation of insulin receptor substrate 1 (IRS-1) on critical sites (e.g., Ser 1101), which in turn blocks the insulin-stimulated tyrosine phosphorylation of IRS-1 and the subsequent binding and activation of phosphatidylinositol (PI) 3-kinase (PI3K). This, in turn, leads to decreased insulin-stimulated glucose transport activity by glucose transporter type 4 (GLUT 4), resulting in decreased insulin-stimulated glycogen synthesis and glucose oxidation. In addition, an increase in DAG in the plasma membrane of skeletal muscles also mediates lipid-induced insulin resistance through the activation of PKC epsilon (PKC ε). This phosphorylates threonine 1150 of the insulin receptor kinase, which, in turn, leads to the inhibition of insulin receptor kinase activity (modified from [20], with permission).

The decreased/incomplete oxidation of fatty acids in mitochondria may further lead to the formation and accumulation of diacylglycerol (DAG), a fatty acid metabolite, in cells [81,82]. A unifying hypothesis (proposed by Shulman et al.) suggests that insulin resistance results from the disturbed mitochondrial oxidation of fatty acids [81,83]. The disturbed mitochondrial oxidation of fatty acids leads to the intracellular accumulation of DAG and other fatty acid-derived molecules [14,83]. By activating several protein kinases, DAG induces insulin resistance in fat cells, hepatocytes, and skeletal muscle cells [14,46,75,84,85,86,87] (Figure 3). These protein kinases, in turn, inhibit insulin signaling by blunting intracellular insulin-stimulated insulin receptor substrate 1-associated PI3-K activity [14,46,75,84,85,86] (Figure 3). This leads to, among other things, the inhibition of insulin-stimulated translocation of the glucose transporter (GLUT-4) to the plasma membrane and decreased glucose uptake in skeletal muscles [75,86]. Thus, the accumulation of ectopic fat and especially DAG probably forms the molecular basis of lipotoxicity-mediated insulin resistance.

## 7. The Role of Reactive Oxygen Species in the Development of Insulin Resistance During Overnutrition

The failing heart in the dysregulated metabolic state of (pre)diabetes and obesity is flooded with excess fuel (given the constant calorie surplus, hyperglycemia, and hyperlipidemia), whereas the heart muscles of the failing heart are simultaneously unable to convert all excess substrates to mechanical energy [74]. It has been hypothesized that fuel supply exceeding the actual energy demands of the heart may lead to the excessive formation of mitochondrial ROS in the heart [74]. This increase may lead to mitochondrial dysfunction and insulin resistance [74]. Moreover, the in vitro incubation of isolated cardiomyocytes with excess glucose stimulates the nonmitochondrial production of ROS by NADPH oxidase independent of glucose metabolism, whereby this latter process may contribute to insulin resistance within 24 h [88]. Thus, excessive mitochondrial ROS production may mediate insulin resistance in the heart.

Superoxide is a major ROS [89]. It has been found that increased mitochondrial superoxide production is a common feature in four different models of insulin resistance (caused by chronic treatment with insulin, corticosteroids, pro-inflammatory cytokines, or lipids) in adipocytes, myotubes, and mice [90].

**Figure 4:**
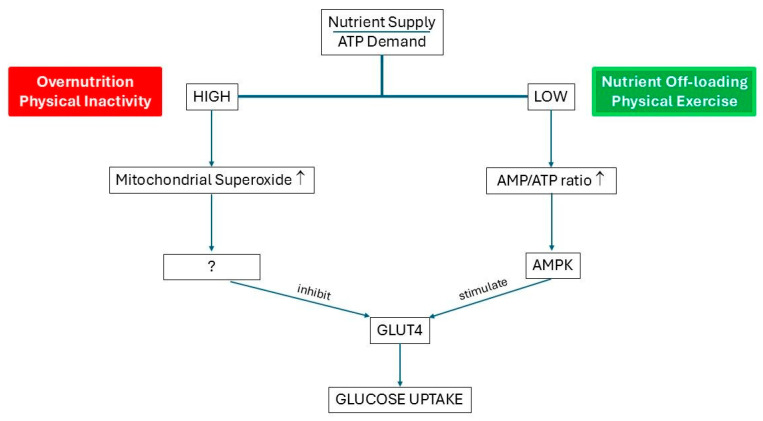
Model explaining how mitochondrial superoxide acts as a nutrient sensor and forms the nexus between intracellular metabolism and the control of insulin action. The ratio of nutrient supply and ATP demand is at the center of this model. When the nutrient supply/ATP demand ratio of the cell is high due to overnutrition/physical inactivity, the cell can rapidly respond to the correct energy surplus by controlling the glucose entry into the cell. A high nutrient supply/ATP demand ratio causes an increased mitochondrial superoxide production in the cells. The increased mitochondrial superoxide production is the signal for the cell to dampen glucose uptake by inhibiting the recruitment of glucose transporter 4 (Glut 4), although the exact mechanism is unknown. As a direct consequence, the glucose uptake by the muscle and fat cells decreases. In contrast, in the opposite situation, when the nutrient supply/ATP demand ratio in the cells is low due to the nutrient off-loading/physical exercise (right), the concomitant increase in the AMP/ATP ratio leads to the activation of AMPK and subsequently the increase in the insulin-mediated glucose uptake by the cells (modified from [90], with permission). Abbreviations: ATP: adenosine triphosphate; AMP: adenosine monophosphate; AMPK: AMP-activated protein kinase, an enzyme that is thought to act as a cellular energy sensor.

Mitochondrial superoxide has been proposed to be the nexus between intracellular metabolism and the control of insulin action: when there is nutrient oversupply, a rise in mitochondrial superoxide production makes cells insulin-resistant, and this decreases insulin-mediated cellular nutrient uptake in key metabolic tissues (liver, skeletal muscles, and heart) [90]. Superoxide-mediated insulin resistance helps the cells to return to an energy-neutral situation by inhibiting glucose uptake [90] (Figure 4). Thus, in the setting of constant exposure to nutrient excess, the development of insulin resistance is regarded as an acquired cellular mechanism trying to protect the heart against substrate overload by oxidizable fuels [91].

**Figure 5:**
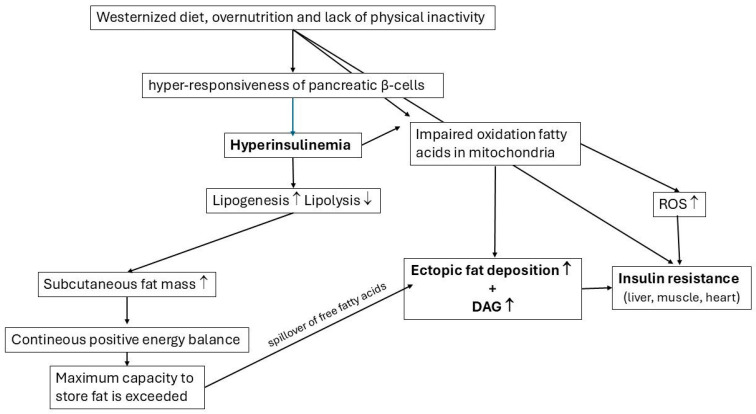
Scheme showing how the Western diet, overnutrition, and physical inactivity are involved in the development of hyperinsulinemia, ectopic fat depositions, excessive ROS production, and insulin resistance. Hyperinsulinemia is caused by the hyper-responsiveness of pancreatic β-cells to a hostile environment and drastic changes in lifestyle (Westernized diet, overnutrition, and a lack of physical activity). The combination of hyperinsulinemia and overnutrition initially stores excess fuel as fat in the subcutaneous fat stores. Consequently, the subcutaneous fat mass initially increases. However, when energy intake is chronically greater than energy expenditure, the maximum capacity of an individual’s subcutaneous fat cells to store excessive fuel as fat is exceeded, and there will be a spillover of free fatty acids into the bloodstream. Excessive fatty acids are then transported to the liver, pancreas, heart, and skeletal muscles and stored in these tissues as ectopic fat. Independent from this mechanism, it has been hypothesized that impaired mitochondrial fat oxidation (due to excessive fuel supply unmatched to (higher than) the actual energy demands) leads to increased ectopic fat depositions. The decreased/incomplete oxidation of fatty acids in mitochondria may further lead to the formation and accumulation of diacylglycerol (DAG), a fatty acid metabolite, and reactive oxygen species (ROS). Both DAG and ROS may (independent of each other) mediate insulin resistance in the liver, muscles, and heart (lipotoxicity). In addition, physical inactivity and glucose toxicity may contribute to insulin resistance.

Figure 5 gives a summary of paragraphs 5–7. It shows how the Western diet, overnutrition, and physical inactivity are involved in the development of hyperinsulinemia, ectopic fat depositions, excessive ROS production, and insulin resistance.

## 8. Insulin Resistance May Protect the Heart from Glucose Overload

**Figure 6:**
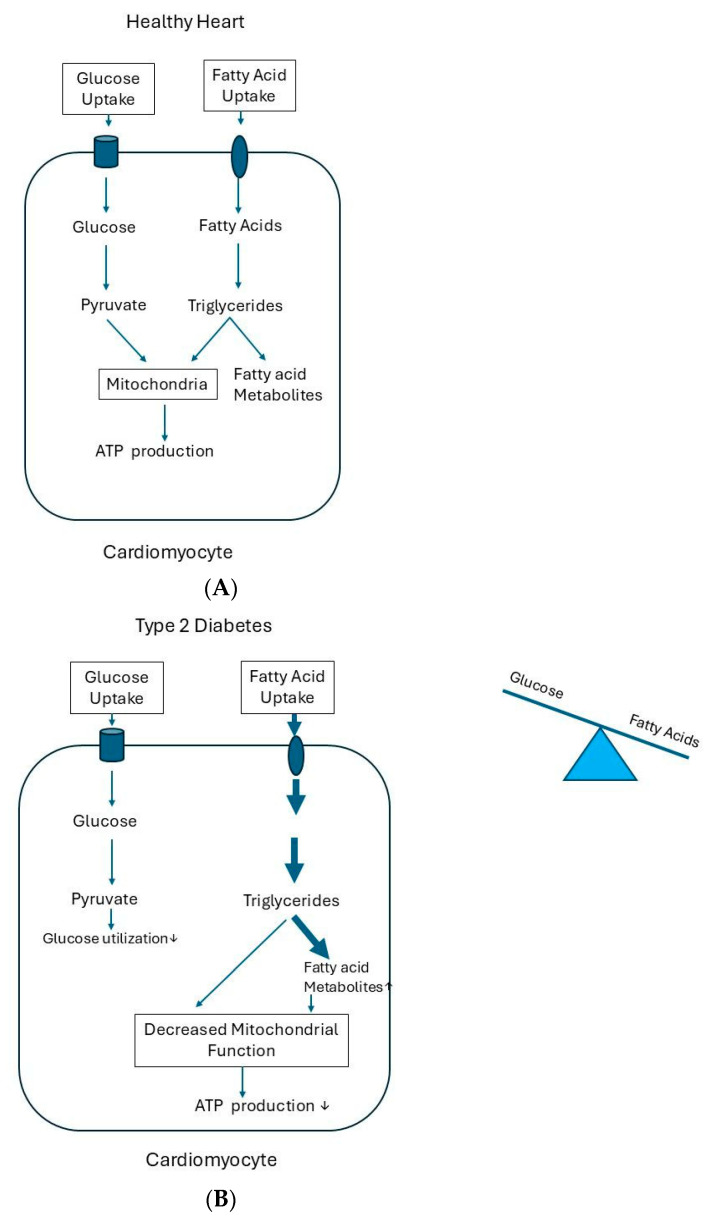
Scheme of energy metabolism in the cardiomyocytes of a healthy heart and in type 2 diabetes. (**A**) In the healthy heart, the rates of glucose and fatty acid uptake by the heart exactly match the rates of substrate utilization. In the healthy state, fatty acids are the major sources of cardiac energy. Most fatty acids undergo rapid oxidation and induce adenosine triphosphate (ATP) production to maintain an adequate cardiac function. Supply and demand of energy-providing substrates is well balanced to meet the energy needs. (**B**) In type 2 diabetes, the substrate availability is increased, and higher levels of fatty acids are preferentially used by the heart as substrates relative to glucose. The development of cardiac insulin resistance causes an increased reliance of the heart on free fatty acid oxidation as an energy source and a simultaneous decrease in glucose oxidation This results in increased fatty acid uptake and fatty acid oxidation in cardiomyocytes. Increased fatty acid oxidation causes reduced ATP production and work efficiency of the heart, possibly as more oxygen is needed per ATP molecule generated and cardiac energy metabolism is reduced. In addition, when fatty acid uptake by cardiomyocytes exceeds the oxidation of fatty acids, this leads to an increased availability of fatty acids for non-oxidative metabolic pathways with a concomitant increase in fatty acid metabolites such as diacylglycerols (DAGs) and ceramides in the cardiomyocytes. Collectively, these fatty acid metabolites cause defective intracellular signaling, create oxidative stress, activate apoptosis, induce mitochondrial dysfunction, and ultimately lead an abnormal cardiac function.

A healthy heart is highly flexible in its choice of substrate, and this is dependent on the prevailing metabolic conditions [92]. In the healthy heart, the rates of glucose and fatty acid uptake exactly match the rates of substrate utilization; the supply and demand of energy-providing substrates are finely tuned to meet the energy needs [92]. Fatty acids are the major sources of cardiac energy. In the healthy heart, 60–80% of cardiac energy is derived from fatty acid oxidation [93]. Most fatty acids undergo rapid oxidation in the healthy heart and induce the production of adenosine triphosphate (ATP) to maintain an adequate cardiac function (Figure 6A). Glucose is the second main fuel source of the heart, accounting for 20–40% of ATP production [94]. The control of substrate supply and demand in the heart normally occurs primarily at the level of the plasma membrane and the mitochondria [74]. The main role of insulin in the heart under normal physiological conditions is the regulation of substrate utilization: promoting glucose uptake and its utilization via glycolysis [95]. By promoting glucose as a cardiac energy substrate, insulin reduces myocardial oxygen consumption and increases cardiac efficiency [95]. In a normal rat heart, when perfused ex vivo at a physiological workload, insulin is not required for the maximal rates of glucose consumption and the maintenance of normal cardiac work at near-physiological conditions [96]. Under these circumstances, the heart can increase glucose uptake independently from insulin to meet the extra energy demands of the increased workload [74,97].

It has been proposed that cardiac insulin resistance develops when there is a chronic positive energy balance, which causes the heart to be overloaded with excess nutrients (fatty acids, glucose, etc.). Cardiac insulin resistance limits glucose overload and protects the heart from the toxic and damaging effects of excess glucose (note: cardiac insulin resistance does not protect against fatty acid overload, see below) [72,73,74]. Several animal and clinical studies support this hypothesis, and these studies demonstrated that insulin resistance lessened glucose overload and toxicity in the stressed heart, while this was accompanied by improved cardiac efficiency [74,98,99,100,101].

The abovementioned metabolic flexibility of the healthy heart is lost in type 2 diabetes, and this is partly attributed to the development of cardiac insulin resistance. Cardiac insulin resistance causes an increased reliance of the heart on free fatty acid oxidation as an energy source and simultaneously decreases glucose oxidation [102] (Figure 6B). This shift in fuel selection likely underlies the cardiac disease risk in type 2 diabetes, since it increases oxygen demand in these circumstances [102]. When positron emission tomography (PET) was used in individuals with type 2 diabetes mellitus to assess the rates of myocardial fatty acid oxidation, an increased fatty acid oxidation was found in the heart [102]. The increased fatty acid oxidation was accompanied by the reduced work efficiency of the heart, which occurred possibly due to the greater oxygen consumption and less ATP production by fatty acid metabolism [102].

Fatty acid uptake exceeding the fatty acid oxidative capacity of the heart may also lead to the accumulation of fat and other cardiotoxic fat intermediates such as DAG and ceramides in the heart [103]. Increasing evidence suggests that the accumulation of these cardiotoxic fat metabolites (like DAG) can further increase cardiac insulin resistance and cause mitochondrial dysfunction, oxidative stress, and the formation of ROS in the heart [103,104]. These processes either directly induce apoptosis and damage of the cardiomyocytes or indirectly lead to cardiac dysfunction by inducing an inflammatory reaction [104,105]. Proton magnetic resonance imaging (1H-MRS) studies have demonstrated an increased intramyocardial adipose tissue content in individuals with metabolic syndrome, impaired glucose tolerance, and type 2 diabetes [106,107].

Interestingly, it has been found that weight loss can reduce cardiac fatty acid uptake, fatty acid oxidation, and excessive intramyocardial content, and this may lead to improvements in left ventricle diastolic function, suggesting that optimizing cardiac energy metabolism may prevent and treat cardiac dysfunction [105,108] (see further paragraphs 10 and 11 below).

## 9. Intensive Glucose Lowering in Type 2 Diabetes and the Risk of Causing Harm

Cardiovascular disease is the major driver of morbidity and mortality in type 2 diabetes [109]. Type 2 diabetes causes excessive cardiovascular morbidity and premature cardiovascular mortality worldwide [110]. In the Swedish nationwide registry data, patients with type 2 diabetes showed less reductions in fatal cardiovascular complications than controls from 1998 to 2014 [98]. In addition, compared with the general Swedish population, persons with type 2 diabetes showed a substantial increase in the overall incidence of all types of cardiovascular disease [111].

For many years, strategies to lower/normalize HbA1c levels have been the cornerstone of type 2 diabetes management. The main glucose-lowering drugs to treat type 2 diabetes were metformin, sulfonylureas, and insulin [112]. Metformin was prescribed to reduce glucose production by the liver, sulfonylureas to increase pancreatic insulin production, and insulin injections to replace insulin deficiency. In the United Kingdom Prospective Diabetes Study (UKPDS), the benefit of intensive glycemic management in type 2 diabetes in microvascular complications was clearly demonstrated [113]. However, after intensive glycemic management, the UKPDS failed to demonstrate a clear/consistent reduction in macrovascular complications and diabetes-related endpoints during the trial [113]. A recent meta-analysis of trials comparing intensive glucose-lowering strategies (N = 51,469 patients with type 2 diabetes) with standard therapy (N = 24,778 patients with type 2 diabetes) was associated with a reduced risk of non-fatal myocardial infarction, retinopathy, and nephropathy [109]. However, there was again (comparable to previous findings in UKPDS) a lack of an effect of intensive glucose-lowering strategies on most macrovascular outcomes [109]. In 2008, the Action to Control Cardiovascular Risk in Diabetes (ACCORD) study showed that an aggressive, intensive glucose-lowering therapy with insulin or thiazolidinedione therapy (with or without insulin) increased mortality, including death from cardiovascular causes in high-risk patients with type 2 diabetes [114]. Post hoc analyses did not support the initial assumption that an increased frequency of hypoglycemia was responsible for the observed increased mortality in patients after an aggressive, intensive glucose-lowering therapy [115]. Moreover, major trials performed in the past comparing intensive with conventional glucose control (ACCORD study; Veterans Affairs Diabetes Trial (VADT)) showed increased cardiovascular or all-cause mortality or both, although statistical significance was only reached in the higher-powered ACCORD study [114,116]. Furthermore, an aggressive, intensive glucose-lowering therapy was associated with weight gain and a higher frequency of hypoglycemia [117].

Aggressive, intensive glucose-lowering therapies have been hypothesized to override the protective effects of insulin resistance against excess nutrients and to increase insulin-mediated metabolic stress/toxicity in the heart and skeletal muscles [74,117]. It was therefore suggested that using high doses of insulin to lower glucose levels could cause direct harm to the heart and the skeletal muscles and induce glucolipotoxicity by exposing cells and tissues to additional nutrients without altering energy expenditure [73,74,117]. High doses of insulin may contribute to the development of metabolic cardiomyopathy and can also induce metabolic myopathy in skeletal muscles, thereby impairing a patient’s ability to exercise [117]. In addition, the administration of high doses of insulin to subjects with the selective insulin resistance of the metabolic PI3K/AKT pathway could lead to the excessive stimulation of the proatherogenic MAPK pathway and thereby increase the risk and incidence of vascular events (Figure 1B) [72,118].

It was long thought that pharmacological therapies reversing insulin resistance could help to reduce the risk of vascular disease in type 2 diabetes. Although metformin is often considered as an insulin sensitizer, metformin per se does not improve peripheral insulin resistance [119]. A recent meta-analysis on the impact of metformin on cardiovascular risk reduction in type 2 diabetes did not provide a clear picture and left its role in this respect undecided [120]. Thiazolidinediones are the only approved pharmacologic agents that specifically reduce insulin resistance [121]. Thiazolidinediones exert their major effects by improving insulin receptor sensitivity, which potentiates the effects of insulin [122]. However, several reports have appeared in the past suggesting that thiazolidinediones may exacerbate heart failure [121,123]. It has been hypothesized that these drugs by improving insulin receptor sensitivity also augment substrate uptake by the diabetic heart, which is already, as discussed above, flooded with excess fats and glucose [91]. As a result, the heart is overwhelmed by oxidizable fuels, leading to heart failure or the worsening of heart health [91]. Moreover, it has been suggested that treatment with thiazolidinediones, when the body is flooded with excess fats and glucose, is potentially dangerous and harmful as it accelerates atherosclerosis [7].

## 10. New Glucose-Lowering Pharmacological Treatments Reduce Dramatic Cardiovascular Events in Type 2 Diabetes

Recent landmark cardiovascular outcome trials in type 2 diabetes have revealed the major cardioprotective actions of glucagon-like receptor-1 agonists (GLP-1 receptor agonists) and certain sodium–glucose transporter-2 (SGLT2) inhibitors [124,125]. A significant reduction in cardiovascular events and mortality was found after treatment with these new drugs [124,125]. A systematic review and meta-analysis based on 169,513 participants with type 2 diabetes showed consistent risk reductions in macrovascular outcomes after treatment with GLP-1 receptor agonists or SGLT2 inhibitors [126]. Interestingly, most of these trials achieved a quick reduction in major adverse cardiovascular events (MACEs) (in months) after the start of GLP-1 receptor agonist or SGLT2 inhibitor therapies in high-risk patients with type 2 diabetes [112]. In addition, GLP-1 receptor agonists and SGLT2 inhibitors also improved renal function and showed renoprotective effects in type 2 diabetes [112].

Treatments of type 2 diabetes with GLP-1 receptor agonists and SGLT2 inhibitors have distinct as well as common mechanisms of action: they both reduce insulin resistance, improve glucose and blood pressure control, and reduce body weight, which all may contribute to a decrease in cardiovascular risk [112].

In comparison to the older antihyperglycemic drugs, SGLT2 inhibitors produce fewer reductions in HbA1c levels when compared with intensive glucose-lowering older agents, suggesting that the marked benefits for major cardiovascular outcomes after treatment with SGLT2 inhibitors extended beyond mere glycemic control [126].

The benefits of GLP-1 receptor agonists and SGLT2 inhibitors on cardiovascular outcomes have been further attributed to various and diverse other effects: the improvement in endothelial function, hemodynamic, anti-hypertensive effects and reduced inflammation, the preferential oxidation of the ketone body β-hydroxybutyrate by the heart in preference of fatty acids, enhanced osmotic diuresis and natriuresis, the improvement in lipid profile, changes in hematocrit and hemoglobin, and direct cardiac benefits [112,126,127,128,129,130]. Although thus a substantial number of effects have been proposed to be involved in the cardiovascular benefits of GLP-1 receptor agonists and SGLT2 inhibitors, it is still not clear how these treatments exactly contribute to their impressive cardiovascular effects. Future studies are still required to clarify this important issue.

This review presents an alternative hypothesis to explain the positive effects on cardiovascular outcomes in type 2 diabetes after treatment with bariatric surgery, GLP-1 receptor agonists, or SGLT2 inhibitors. We propose that the beneficial effects on cardiovascular outcomes in type 2 diabetes after these three treatments are critically dependent on the reduction in calorie intake/loss of calories and the subsequent weight loss and improvement in insulin receptor sensitivity as a consequence of nutritional off-loading. The nutritional off-loading normalizes energy balance, lowers circulating glucose and free fatty acid levels, results in a reduction in ectopic fat depositions and ROS activity, and improves mitochondrial function. We hypothesize that this may be a central mechanism that is responsible for the observed dramatic reduction in cardiovascular disease outcomes and the reversal of insulin resistance in type 2 diabetes after these three treatments (see next paragraphs for more details). However, this new hypothesis keeps the option open that other mechanisms (such as hemodynamic effects, ketone body metabolism, direct myocardial and renal mechanisms, endothelial effects, and anti-inflammatory effects) may contribute to the beneficial cardiovascular effects after treatment with bariatric surgery, GLP-1 receptor agonists, or SGLT2 inhibitors.

## 11. Bariatric Surgery, SGLT2 Inhibitors, GLP-1 Receptor Agonists, Induction of a Negative Energy Balance, and Improvement in Cardiovascular Outcomes

Any intervention achieving a negative energy balance over an extended period results in weight loss [131]. Reduced calorie intake by diet and increased physical exercise have been recommended for years for the management of type 2 diabetes. However, diet and increased physical exercise often fail in daily clinical practice to achieve a long-term negative energy balance in most individuals with type 2 diabetes [132]. Moreover, to date, there is no available evidence showing that reduced calorie intake by diet and/or increased physical exercise reduce cardiovascular disease and mortality [132,133].

Bariatric surgery is currently the most effective strategy to induce a negative energy balance and to achieve weight loss [134,135]. It causes substantial and sustained weight loss [136]. Immediately after bariatric surgery, patients are instructed to consume a diet within the range of 500–800 kcal/day, while intake postoperatively gradually increases to an average 1000–1500 kcal/days for maintenance [135]. A negative energy balance after bariatric surgery is primarily achieved by drastically reduced food intake and malabsorption, while resting and total expenditure may be decreased due to weight loss and metabolic adaptations [137]. A meta-analysis reported that bariatric surgery decreased mean energy intake by 1050 kcal/day when compared with the baseline energy intake [138]. In six months, bariatric surgery significantly induced nutrient off-loading and reduced ectopic fat depositions in the liver, pancreas, visceral fat, and, to a lesser extent, epicardial adipose tissue, improving metabolic health [139]. However, although bariatric surgery is thus highly effective, its benefits should be weighed against potential complications: significant, procedure-specific surgical, metabolic, and nutritional long-term adverse effects/complications.

GLP-1 receptor agonist treatment promotes a negative energy balance by suppressing appetite, enhancing satiety, and delaying gastric emptying, which altogether lead to reduced caloric consumption and glucose levels and increased weight loss [140]. A recent review reported that treatment with GLP-1 receptor agonists or GIP/GLP-1 receptor agonists reduced daily calorie intake by 16–39% [141].

Treatments with SGLT-2 inhibitors induce a negative energy balance by promoting glucose excretion in the urine, which creates an energy deficit [142]. This triggers catabolism by promoting fat breakdown and the production of ketone bodies as an energy source, while also reducing fat mass [142]. By promoting the excretion of 60–80 g of glucose per day through the urine, treatments with SGLT2 inhibitors lead to a significant loss of 240–320 calories per day [143]. Although during treatment with SGLT2 inhibitors glycosuria is persistent over time, weight loss during follow-up is much less than would be expected based on daily calorie loss through the urine [143]. The lower overall weight loss during treatment with SGLT2 inhibitors is the result of metabolic adaptations like compensatory hyperphagia [143].

Ten years ago, Nolan et al. predicted that glucose-lowering approaches that simultaneously off-load nutrients to the myocardium and vascular endothelium had the greatest potential to improve the cardiovascular outcomes of insulin-resistant patients with type 2 diabetes [72]. Compared to treatment with GLP-1 receptor agonists or SGLT2 inhibitors, bariatric surgery leads to better glucose control, greater remission of type 2 diabetes, and improvements in cardiovascular disease and other type 2 diabetes outcomes [118,119,120,121,122]. This suggests that the degree of negative energy balance and nutrient off-loading play a major role in improving cardiovascular disease outcomes after these three interventions.

The cardiovascular outcomes after bariatric surgery, GLP-1 receptor agonist treatment, or SGLT2 inhibitor treatment markedly contrast those of past trials in which variable combinations of sulfonylureas, metformin, thiazolidinediones, and/or insulin were used to treat patients with type 2 diabetes [112]. As previously discussed, (combinations of) these antihyperglycemic drugs failed to reduce cardiovascular events despite significantly lowering HbA1c levels and reducing microvascular complications [112]. However, in contrast to bariatric surgery and the new glucose-lowering pharmacological treatments, none of the (combinations of) traditionally used drugs lead to significant long-term weight loss and nutrient off-loading.

## 12. Effects of Bariatric Surgery, SGLT2 Inhibitors, and GLP-1 Receptor Agonists on Insulin Sensitivity

More than 30 years ago, Pories et al. were the first to show that bariatric surgery produced the durable and complete control of type 2 diabetes [144]. Interestingly, significant improvements in insulin sensitivity occurred in the initial days and weeks after bariatric surgery while there was hardly any weight loss [145,146,147]. This suggests that it was not the loss of body weight but other mechanisms that were primary responsible for the dramatic and almost instantaneous reversal of insulin resistance after bariatric surgery [135]. The abrupt off-loading of excess nutrients and the subsequent reduction in ectopic fat depositions after bariatric surgery have been proposed to be important factors for explaining the reversal of insulin resistance after bariatric surgery [147]. The minimization of ectopic fat depots may promote insulin sensitivity in skeletal muscles after bariatric surgery [148]. A striking reduction in intra-myocellular triglycerides (ectopic fat) in skeletal muscle biopsies was observed at 3 and 9 months after bariatric surgery [147]. A reduction in intra-myocellular triglycerides in skeletal muscles after bariatric surgery was proposed to be most likely caused by a decreased free fatty acid supply to muscles, the increased rates of triglyceride hydrolysis in muscles, and the increased free fatty oxidation in muscles [147]. A significant improvement in all studied types of ectopic fat depots (liver, pancreas, visceral adipose tissue, and heart) was observed at 32 months after bariatric surgery [149]. Thus, the off-loading of excess nutrients after bariatric surgery eliminates ectopic fat deposits in insulin-responsive tissues, thereby resolving lipotoxicity and reversing insulin resistance [147]. A decrease in systemic insulin resistance and an improvement in the diastolic dysfunction of the heart were observed at 6 months after bariatric surgery [139]. These changes were accompanied by a significant decrease in epicardial fat (which is also a form of ectopic fat) in the heart [139]. It was therefore proposed that the improvement in diastolic dysfunction of the heart after bariatric surgery was the direct consequence of the decrease in epicardial fat, which leads to improved insulin sensitivity [147,150]. Lowered insulin levels, increased adiponectin levels, increased GLP-1 levels, weight loss, and decreased inflammation are other factors that may contribute to improved insulin sensitivity after bariatric surgery [151].

GLP-1 receptor agonist treatment (and combined GIP/GLP-1 receptor agonist treatment) may also promote the redistribution of fat from visceral to subcutaneous depots, enhance fatty acid oxidation, and thereby minimize ectopic fat depositions and improve overall metabolic health [140,152]. Several studies suggest that excess liver fat and visceral fat accumulation are linked to insulin resistance [153]. GLP-1 receptor agonists reduced (ectopic) liver fat and drastically lowered (ectopic) visceral fat in adults with type 2 diabetes [154]. Moreover, further evidence showed that GLP-1 receptor agonists can improve insulin sensitivity by attenuating oxidative stress in type 2 diabetes [155,156]. Interestingly, as previously found for bariatric surgery, GLP-1 receptor agonists were found to improve liver insulin sensitivity (measured by HOMA) and decrease fasting and postprandial glucose prior to significant weight loss [157]. GLP-1 receptor agonists further suppress glucagon secretion, which may further contribute to improved insulin sensitivity [158]. GLP-1 receptor agonist treatment results in a significant decrease in glycated hemoglobin, and this is another effect that may contribute to improvements in insulin sensitivity [159]. In addition, GLP-1 receptor agonists can improve insulin sensitivity by lowering the inflammatory responses [160].

A recent meta-analysis showed that SGLT2 inhibitors reduce insulin resistance [150]. SGLT2 inhibitors improve insulin resistance by increasing caloric disposition and reducing glucose levels through increased urinary glucose excretion [150,161]. SGLT2 inhibitors further improve insulin sensitivity by reducing ectopic fat storage in the liver, the heart (epicardial fat), and around internal organs in the abdomen (visceral fat) [161,162,163,164,165,166,167,168]. They could further improve insulin sensitivity by reducing oxidative stress [161]. While reductions in ectopic fat likely contribute to the long-term metabolic effects of SGLT2 inhibitors, this cannot adequately explain the rapid cardiovascular benefits observed in landmark clinical cardiovascular outcome trials. The rapid onset of cardiovascular benefits, specifically reductions in heart failure hospitalizations, suggests that other, more immediate hemodynamic and metabolic mechanisms induced by SGLT2 inhibitors are responsible for these effects [169]. Multiple preclinical and human studies have suggested that SGLT2 inhibitors also have anti-inflammatory effects, and this may be another mechanism by which SGLT2 inhibitors contribute to an improvement in insulin sensitivity [170]. Table 2 summarizes and compares the effects of bariatric surgery, GLP-1 receptor agonists, and SGLT2 inhibitors on body weight, several parameters of metabolism, cardiovascular disease, and mortality in individuals with type 2 diabetes. It shows that bariatric surgery, GLP-1 receptor agonists, and SGLT2 inhibitors have many similarities in terms of treatment targets and effects but that they also significantly differ in several respects. To date, the effectiveness of bariatric surgery, GLP-1 receptor agonists, and SGLT2 inhibitors in type 2 diabetes has rarely been compared [171]. When these three treatment options were compared in individuals with type 2 diabetes and obesity, the bariatric surgery group showed the highest weight loss and the best metabolic outcomes at the 12-month follow-up, but bariatric surgery was also the most expensive treatment in the short term [171]. Comparative longitudinal studies with a longer follow-up are needed to better assess the long-term efficacy and cost-effectiveness of bariatric surgery and GLP-1 receptor agonist and SGLT2 inhibitor treatments.

## 13. Concluding Remarks

**Figure 7:**
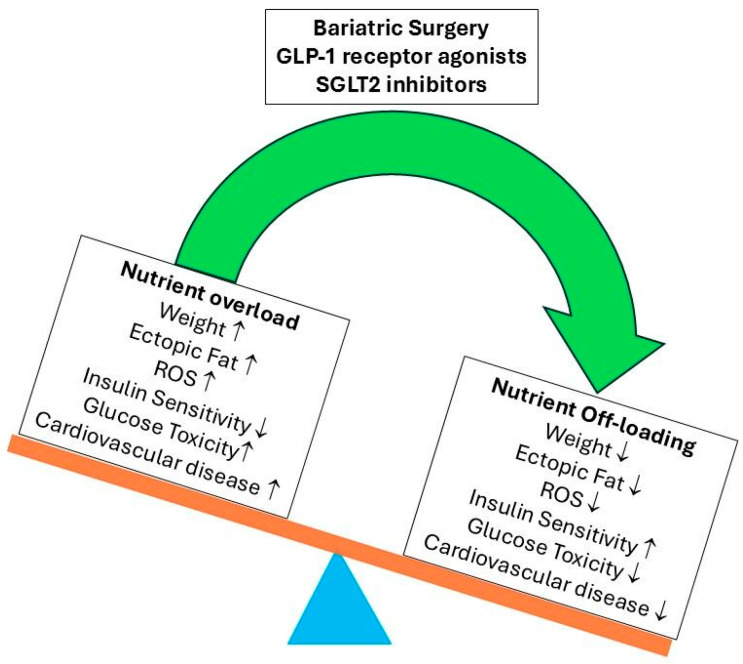
Effects of bariatric surgery, GLP-1 receptor agonists, and SGLT2 inhibitors on cardiovascular disease in type 2 diabetes. Nolan et al. previously predicted that glucose-lowering approaches that simultaneously off-load nutrients to the myocardium and vascular endothelium had the greatest potential to improve the cardiovascular outcomes of insulin-resistant patients with type 2 diabetes [72]. The treatment with bariatric surgery, GLP-1 receptor agonists, or SGLT2 inhibitors normalizes whole-body energy balance by nutrient off-loading: this reduces body weight, ectopic fat depositions, intracellular diacylglycerol (DAG), and reactive oxygen species (ROS), which improves insulin sensitivity and decreases glucose toxicity. All these effects contribute to the dramatic reduction in cardiovascular disease in type 2 diabetes observed after treatment with bariatric surgery, GLP-1 receptor agonists, or SGLT2 inhibitors (see the main text for more details).

In recent years, insulin resistance in type 2 diabetes has been associated with cardiovascular disease, including impaired heart function and the development of atherosclerosis. The Westernized diet, chronic nutritional overload, hyperinsulinemia, and physical inactivity are the major etiological factors driving the development of insulin resistance. It has been hypothesized that, in an obesogenic environment, the development of insulin resistance serves to defend the body against toxic fuel overload, hyperinsulinemia-induced injury, and metabolic stress. For many years, strategies that lower/normalize HbA1c levels using metformin, sulfonylureas, and insulin have been the primary cornerstone of type 2 diabetes management. However, intensive glucose-lowering strategies using these traditional drugs produced disappointing results and did not lead to unequivocal improvements in (most) macrovascular outcomes. In contrast, recent landmark cardiovascular outcome trials in type 2 diabetes demonstrated significant improvements in cardiovascular disease following treatment with SGLT2 inhibitors or GLP-1 receptor agonists. Bariatric surgery resulted in even greater improvements in cardiovascular disease outcomes in individuals with type 2 diabetes. Ten years ago, Nolan et al. predicted that glucose-lowering approaches that simultaneously off-load nutrients to the myocardium and vascular endothelium had the greatest potential to improve cardiovascular outcomes in type 2 diabetes [72]. This review supports this view and hypothesizes that treatments with bariatric surgery, GLP-1 receptor agonists, or SGLT2 inhibitors that result in glucose lowering, nutritional off-loading, and a reduction in ectopic fat depositions normalize whole-body energy balance. This plays a key role in the observed dramatic reduction in cardiovascular disease outcomes and the reversal of insulin resistance in type 2 diabetes after bariatric surgery and GLP-1 receptor agonist and SGLT2 inhibitor treatments (Figure 7).

Although it provides a plausible explanation for the observed dramatic reduction in cardiovascular disease outcomes and the reversal of insulin resistance in type 2 diabetes after bariatric surgery and GLP-1 receptor agonist and SGLT2 inhibitor treatments, a limitation of the nutritional off-loading hypothesis is that it does not capture metabolic heterogeneity between individuals (i.e., metabolic responses to the same intervention can significantly vary between human individuals). Moreover, it should be recognized that the nutritional off-loading hypothesis has at present a high reliance on associated clinical data, rather than direct causal evidence, and this should be further addressed in future research.

## Figures and Tables

**Table 1:** Acquired and secondary causes of insulin resistance.

Hyperinsulinemia [16,17,18]
Nutrient excess [19]
Adiposity related to ectopic fat depositions and overflow from subcutaneous fat stores [20]
Aging [21]
Physical inactivity [22]
Glucose toxicity [23]
Lipotoxicity (from excess circulating free fatty acids) [23]
Stress (due to excess counter-regulatory hormones such as cortisol, catecholamines, growth hormone, and glucagon) [24]
Infection [25]
Puberty [26]
Pregnancy [27]
Starvation [28]
Low-grade inflammation (by macrophages, Tumor Necrosis Factor-α, Interleukin-1β, etc.) [29,30]
Immune-mediated (anti-insulin antibodies, anti-insulin receptor antibodies) [31,32]
Medications (glucocorticoids, growth hormone, oral contraceptives) [33,34,35]

**Table 2:** Comparison type 2 diabetes of treatment effects of bariatric surgery, GLP-1 receptor agonists, and SGLT2 inhibitors on body weight, several parameters of metabolism, cardiovascular disease, and mortality.

	Bariatric Surgery	GLP-1 Receptor Agonists	SGLT2 Inhibitors
Effect on body weight	↓↓↓	↓↓	↓
Effect on ectopic fat	↓	↓	↓
Fasting insulin levels	↓↓	↓	↓
Insulin resistance	↓	↓	↓
Glucose uptake (insulin-mediated)	↑	↑	↓
HbA1C	↓↓	↓↓	↓
Hepatic glucose production	↓	↓	↑
Ketone body production	↓ a	↓	↑
Blood pressure	↓	↓	↓
Blood lipids	↓	↓	↔/↓ b
Risk of ASCVD	↓	↓	↔/↓ c
Risk of heart failure	↓	↑↔↓ d	↓↓
Risk of stroke	↓	↓↓	↔
Diuresis and natriuresis	↑	↑ (acutely)	↑
Urinary glucose excretion	↔	↔	↑
Renoprotection	↑	↔ e	↑
Cardiovascular death	↓	↓	↓

Effects presented in Table 2 are a summary of results collected from previously published papers [125,161,171,172,173,174,175,176,177,178,179,180,181,182] and studies discussed in this review. a: initially in first weeks ↑; after 6 months ↓. b: SLGT2 inhibitors modestly decrease triglycerides and increase HDL cholesterol but simultaneously increase LDL cholesterol. c: protection from ASCVD with SGLT2 inhibitors may be restricted to secondary prevention. d: GLP-1 receptor agonists may decrease the risk of new-onset HF and may have a neutral effect on HF events in those with heart failure with preserved ejection fraction (HFpEF), but they may increase the risk of adverse events in individuals with heart failure with reduced ejection fraction (HFrEF). e: GLP-1 receptor agonists predominantly reduce albuminuria with a mostly neutral effect on estimated glomerular filtration rate (eGFR); among those with previous chronic kidney disease (CKD), GLP-1 receptor agonists may also slow the decline in eGFR. ASCVD: Atherosclerotic Cardiovascular Disease. ↓ Decreased; ↔ Neutral; ↑ Increased.

## Data Availability

No new data were created or analyzed in this study. Data sharing is not applicable to this article.
